# Endobronchial sialolipoma. Case report

**DOI:** 10.1186/s13000-021-01074-7

**Published:** 2021-02-21

**Authors:** Severino Rey Nodar, Verónica García Yllán, Nohelia Rojas Ferrer, Onay Solis, Hugo D. Boccara

**Affiliations:** 1 Pathology Department, Manises Hospital, Synlab Iberia, Valencia, Spain; 2 FORESC (Foundation for Sciences and Research), Florida, USA

**Keywords:** Case report, Sialolipoma, Salivary gland tumour, Endobronchial tumour

## Abstract

**Background:**

A 52-year-old woman presented with shortness of breath and cough. An endobronchial sialolipoma was found at the left entrance of the main bronchus. Sialolipoma is an exceedingly rare type of lipoma reported of the minor salivary glands, especially within the bronchus.

**Case presentation:**

A 52-year-old woman presented with shortness of breath and cough with 6 months´ evolution. Endobronchial endoscopy revealed a tumour at the left entrance of the main bronchus. The entire removal of the tumour was removed using a cryoprobe device. Pathological examination showed a tumour consistent with the diagnosis of sialolipoma due to the presence of mature adipose cells blended with acinar, ductal, basal, and myoepithelial cells. The patient had a favourable outcome.

**Conclusion:**

The infrequent tracheobronchial presentation of this tumour can be challenging for correct diagnosis.

## Background

Tracheobronchial tree obstructions can be caused by endobronchial tumours despite their clinical and biological behaviour. Tumour removal is the primary treatment to support airway function and to improve the respiratory status.

Benign tumours of the endobronchial tree are rare, with endobronchial lipomas are being the least common.

Lipomas are common benign mesenchymal tumours originated in the human body [1, 2], and Sialolipoma is a remarkably rare type of lipoma found in the major and minor salivary glands that consisting of mature adipocytes intermingled with glandular tissue [3].

Nagao T et al. [3], proposed the designation of sialolipoma to characterize this new category of a benign lipomatous tumour occurring in the salivary glands (mainly in the parotid gland) or in any oral or maxillofacial site, consisting of islands of epithelial salivary gland elements (acinar and ductal structures) enclosed in mature adipose tissue and bounded by a thin fibrous tissue.

This tumour typically occurs in adults (90%), with fewer cases reported in infants [3, 4].

These tumour can also developed inside the bronchus though there are few endobronchial cases reported in the literature. Indeed, despite a wide investigation, we only found a case reported by Kajiwara N et al. [5]. However, under-reporting is possible.

The aetiopathogenesis is unclear.

## Case presentation

**Fig. 1:**
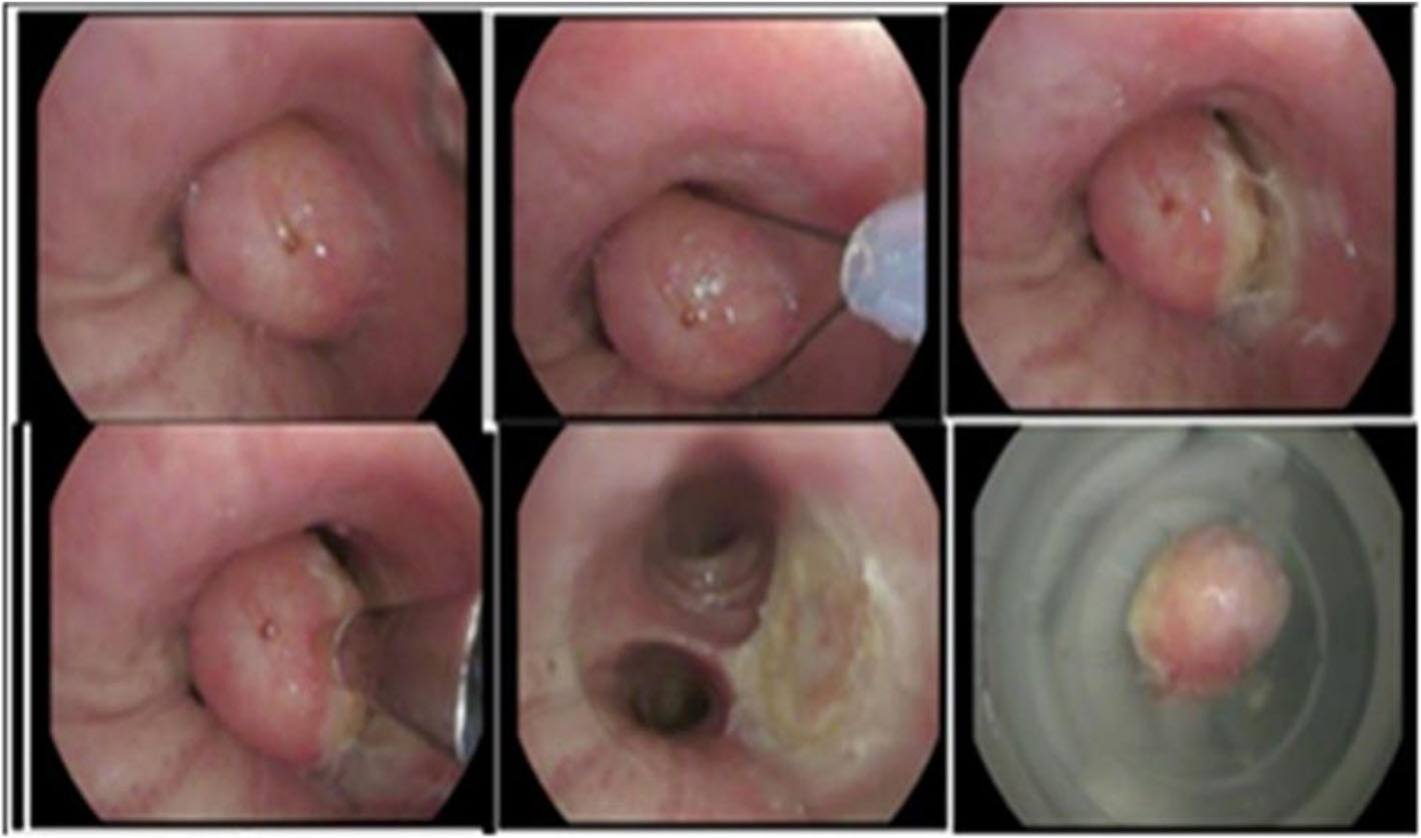
The inside of the left main bronchus during the tumour removal procedure. A round shape, smooth surface, solid and whitish-pink tumour was removed

A 52-year-old woman presented complaining of shortness of breath and cough with 6 months of evolution. The patient reported the use of tobacco for 25 years and a medical history of hypertension. A chest X-ray was performed revealing no lung lesions. There were no relevant findings during a physical examination. Endobronchial endoscopy showed scarce serous secretions in the trachea and major bronchi. An endobronchial tumour was found at the entrance of the left main bronchus shown. A flexible cryoprobe was used for tumour extraction and argon plasma coagulation was applied to control bleeding (Fig. 1).

The patient was discharged a few days after the procedure, and she remains well to date.

**Fig. 2:**
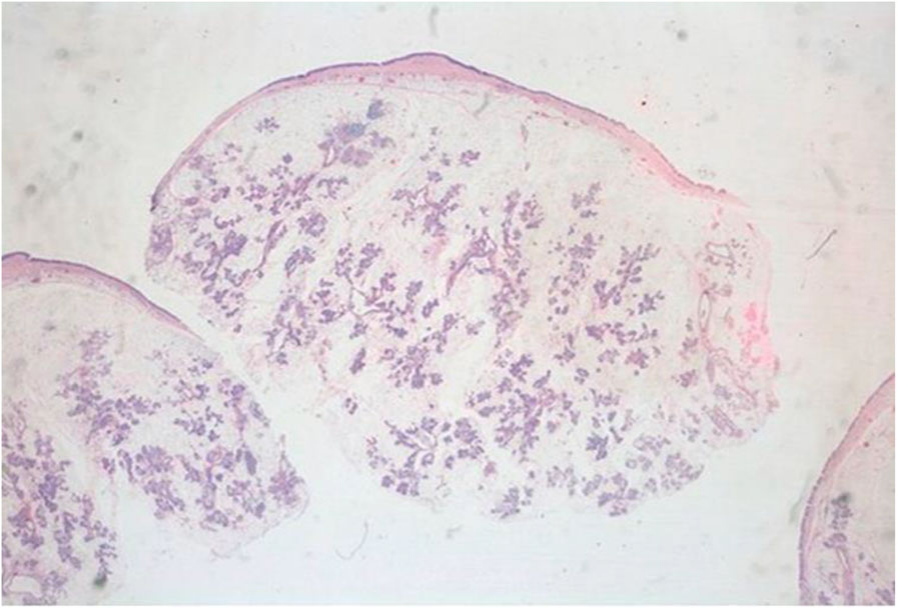
A mass mainly composed of adipose tissue and epithelial elements. (H&E. 4X)

**Fig. 3:**
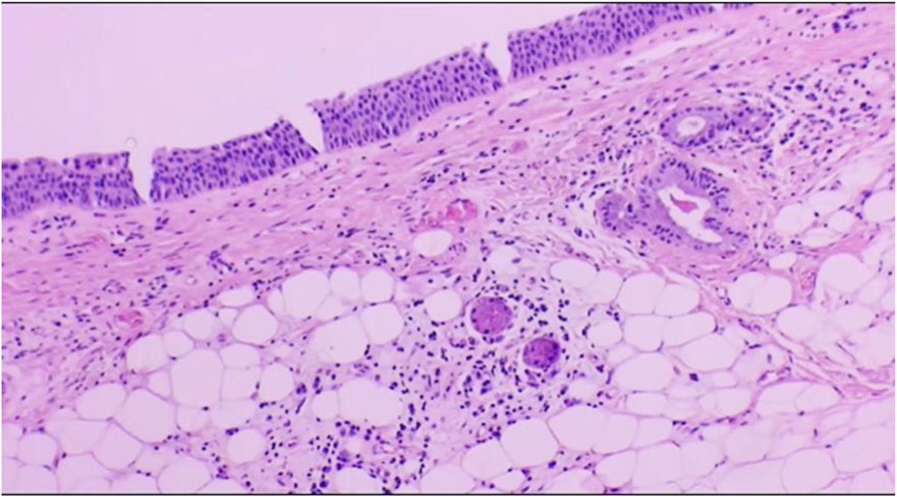
A lipomatous tumour lined by respiratory cylindric epithelial cells, bronchial type. (H&E. 10X)

**Fig. 4:**
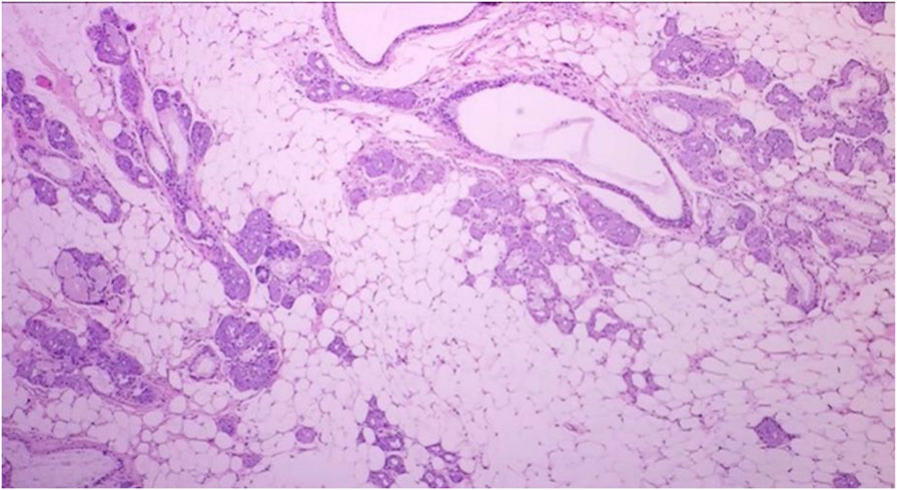
Most of the lesion consists of adipose cells compared to epithelial elements. (H&E. 20X)

**Fig. 5:**
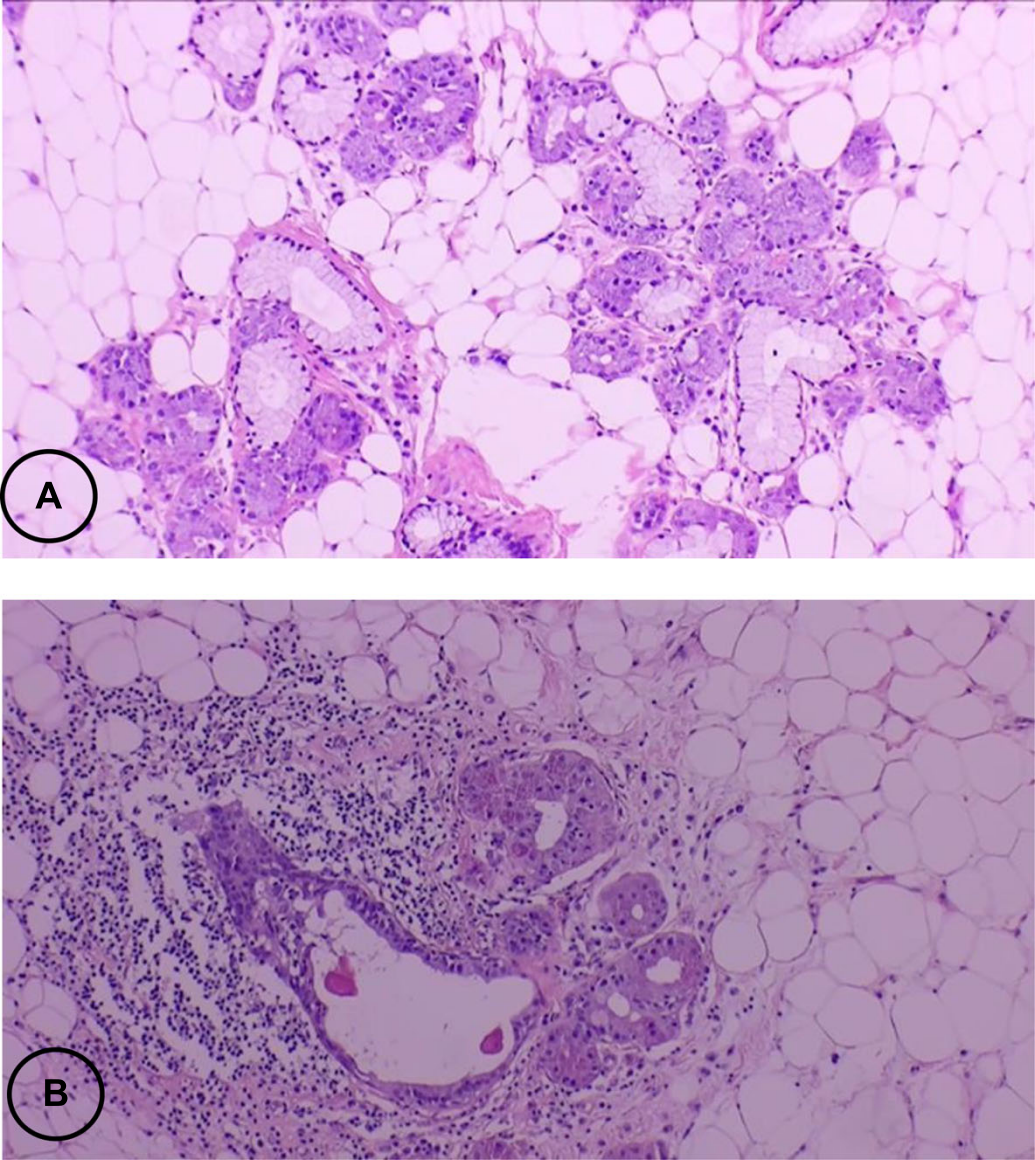
**a** A tumour consisting of mature adipose tissue intermingled with uniform epithelial islets of normal duct-acinar units of salivary gland parenchyma type, without any atypia, **b** Prominent lymphoid infiltrates surrounds the area. (H&E. 40X)

Gross examination showed a round shape, smooth surface, solid and whitish-pink tumour histologically consistent with a sialolipoma due to the presence of mature adipose cells blended with acinar, ductal, basal, and myoepithelial cells (Figs. 2, 3, 4 and 5).

## Discussion and conclusions

Lipomas are common benign subcutaneous or deep soft tissue tumours composed of lobules of mature adipose tissue. They can occur at any age, with most diagnoses occurring in the adults with some cases reported in infants [1, 2].

Sialolipoma is a rare salivary gland lesion characterized by benign salivary gland parenchyma interspersed with mature adipose tissue.

One of the hypotheses regarding the pathogenesis correlates the tumour with salivary gland dysfunction and the substitution of normal glandular tissue by mature adipose tissue, atrophy of the glandular structures, chronic ductal epithelial changes (oncocytic metaplasia), fibrosis, and lymphocytic infiltration [3].

Histological examination in our case revealed a tumour consisting of mature adipose cells mixed with acinar, ductal, basal, and myoepithelial cells inside the left main bronchus. Therefore, the diagnosis of endobronchial sialolipoma was established. A diagnosis of endobronchial lipoma diagnosis was roled out because this entity is characterized by a uniform appearance of mature univacuolated adipocytes of uniform size and shape, without intermingled epithelial ductal-acinar components. In contrast, very few cases inside the bronchus have been reported worldwide [5].

Sialolipoma can be found within both major and minor salivary glands [6–8]. In contrast, very few cases inside the bronchus have been reported worldwide [5].

Regarding differential diagnosis, bronchial lipomas, neuroectodermic tumours, hamartomas, carcinomas, and other tumours must be considered, and definitive diagnosis may be a challenge. Thus, the possibility of an endobronchial tumour should be considered for patients with respiratory symptoms, asthmatic patients who are not responsive to bronchodilators, and those with a medical history of recurrent pneumonia.

Chest tomography and magnetic resonance imaging can show exhaustive details of the tumour characteristics, determining the base to be narrow or broad and extension across the cartilaginous rings, and demonstrating extramural invasion, or the presence of distal lesions. Additionally, the tumour position, size, surface appearance and degree of mobility can be accurately confirmed [9].

In the case we present, endobronchial endoscopy was used for detection of the lesion.

Bronchoscopic resection is considered the first choice of treatment; however, surgical removal is indicated in cases associated with malignant tumour, in patients with destructive peripheral lung disease, with extrabronchial tumour, with evident technical difficulties for bronchoscopic procedures [10].

## Conclusions

Sialolipoma is a recently described variant of lipoma. The rare clinical presentation of this tumour can be challenging for a correct diagnosis because of the need to rule out the much more frequent malignancies. Computed tomography or magnetic resonance are the main procedures employed; however, definitive diagnosis is achieved by bronchoscopy and biopsy of the tumour. Therefore, the treatment of choice is endoscopic resection.

## Data Availability

Not applicable. Our manuscript does not contain any numerical data.
